# RyR2-Mediated Ca^2+^ Release and Mitochondrial ROS Generation Partake in the Synaptic Dysfunction Caused by Amyloid β Peptide Oligomers

**DOI:** 10.3389/fnmol.2017.00115

**Published:** 2017-04-25

**Authors:** Carol D. SanMartín, Pablo Veloso, Tatiana Adasme, Pedro Lobos, Barbara Bruna, Jose Galaz, Alejandra García, Steffen Hartel, Cecilia Hidalgo, Andrea C. Paula-Lima

**Affiliations:** ^1^Department of de Neurology and Neurosurgery, Clinical Hospital Universidad de ChileSantiago, Chile; ^2^Biomedical Neuroscience Institute, Faculty of Medicine, Universidad de ChileSantiago, Chile; ^3^Institute for Research in Dental Sciences, Faculty of Dentistry, Universidad de ChileSantiago, Chile; ^4^Centro Integrativo de Biología y Química Aplicada, Universidad Bernardo O HigginsSantiago, Chile; ^5^Anatomy and Developmental Biology Program, Institute of Biomedical Sciences, Center of Medical Informatics and Telemedicine and National Center for Health Information Systems, Faculty of Medicine, Universidad de ChileSantiago, Chile; ^6^Physiology and Biophysics Program, Institute of Biomedical Sciences, Faculty of Medicine, Universidad de ChileSantiago, Chile

**Keywords:** endoplasmic reticulum, reactive oxygen species, mitochondrial calcium, antioxidants, Alzheimer’s disease

## Abstract

Amyloid β peptide oligomers (AβOs), toxic aggregates with pivotal roles in Alzheimer’s disease, trigger persistent and low magnitude Ca^2+^ signals in neurons. We reported previously that these Ca^2+^ signals, which arise from Ca^2+^ entry and subsequent amplification by Ca^2+^ release through ryanodine receptor (RyR) channels, promote mitochondrial network fragmentation and reduce RyR2 expression. Here, we examined if AβOs, by inducing redox sensitive RyR-mediated Ca^2+^ release, stimulate mitochondrial Ca^2+^-uptake, ROS generation and mitochondrial fragmentation, and also investigated the effects of the antioxidant *N*-acetyl cysteine (NAC) and the mitochondrial antioxidant EUK-134 on AβOs-induced mitochondrial dysfunction. In addition, we studied the contribution of the RyR2 isoform to AβOs-induced Ca^2+^ release, mitochondrial Ca^2+^ uptake and fragmentation. We show here that inhibition of NADPH oxidase type-2 prevented the emergence of RyR-mediated cytoplasmic Ca^2+^ signals induced by AβOs in primary hippocampal neurons. Treatment with AβOs promoted mitochondrial Ca^2+^ uptake and increased mitochondrial superoxide and hydrogen peroxide levels; ryanodine, at concentrations that suppress RyR activity, prevented these responses. The antioxidants NAC and EUK-134 impeded the mitochondrial ROS increase induced by AβOs. Additionally, EUK-134 prevented the mitochondrial fragmentation induced by AβOs, as previously reported for NAC and ryanodine. These findings show that both antioxidants, NAC and EUK-134, prevented the Ca^2+^-mediated noxious effects of AβOs on mitochondrial function. Our results also indicate that Ca^2+^ release mediated by the RyR2 isoform causes the deleterious effects of AβOs on mitochondrial function. Knockdown of RyR2 with antisense oligonucleotides reduced by about 50% RyR2 mRNA and protein levels in primary hippocampal neurons, decreased by 40% Ca^2+^ release induced by the RyR agonist 4-chloro-m-cresol, and significantly reduced the cytoplasmic and mitochondrial Ca^2+^ signals and the mitochondrial fragmentation induced by AβOs. Based on our results, we propose that AβOs-induced Ca^2+^ entry and ROS generation jointly stimulate RyR2 activity, causing mitochondrial Ca^2+^ overload and fragmentation in a feed forward injurious cycle. The present novel findings highlight the specific participation of RyR2-mediated Ca^2+^ release on AβOs-induced mitochondrial malfunction.

## Introduction

Alzheimer’s disease (AD) is currently considered a Ca^2+^-driven pathology (Berridge, 2013; Area-Gomez and Schon, 2017; Frazier et al., 2017; Popugaeva et al., 2017). Familiar AD mutations result in enhanced intracellular Ca^2+^ release via ryanodine receptor (RyR) and inositol 1,4,5-trisphosphate receptor (IP_3_R) channels (Popugaeva and Bezprozvanny, 2013). Of note, cytoplasmic Ca^2+^ levels are higher than normal in familial AD, presumably due to anomalous Ca^2+^ release from the endoplasmic reticulum (ER) (Popugaeva and Bezprozvanny, 2013). Furthermore, primary hippocampal neurons from mice carrying a mutation in the amyloid precursor protein (APP) display increased intracellular Ca^2+^ levels (Koizumi et al., 1998).

We reported previously that amyloid β peptide oligomers (AβOs) induce anomalous Ca^2+^ signals in primary hippocampal neurons; these signals arise initially from Ca^2+^ entry through *N*-Methyl-D-aspartate (NMDA) receptors and are subsequently amplified via RyR channels co-stimulated by Ca^2+^ entry signals and the increased ROS levels produced by AβOs (Paula-Lima et al., 2011; SanMartín et al., 2012a). Furthermore, the levels of RyR2, which is the most abundant RyR isoform expressed in the brain (Giannini et al., 1995), are 20% lower in the brain from AD cases compared to controls (Kelliher et al., 1999). Interestingly, the redox-sensitive abnormal Ca^2+^ signals elicited by AβOs significantly decrease RyR2 expression levels in hippocampal neurons (Paula-Lima et al., 2011; Lobos et al., 2016). Moreover, previous work using selective knockdown techniques established that decreasing RyR2/RyR3 expression negatively affects hippocampal-dependent memory processes (Galeotti et al., 2008), whereas intrahippocampal brain derived neurotrophic factor (BDNF) injection (Adasme et al., 2011) and spatial memory training (Zhao et al., 2000; Adasme et al., 2011) increase RyR2 channel expression. Accordingly, it becomes important to investigate whether the RyR2 isoform is particularly involved in the alterations in intracellular Ca^2+^ signaling and homeostasis induced by AβOs in hippocampal neurons.

The persistent but low-amplitude redox-sensitive RyR-mediated Ca^2+^ signals elicited by AβOs prevent the spine remodeling prompted by BDNF, and provoke mitochondrial network fragmentation (Adasme et al., 2011; Paula-Lima et al., 2011). The ER and mitochondria exhibit physical and functional associations in neurons (Zampese et al., 2011). Indeed, effective mitochondrial Ca^2+^ uptake requires the proximity of mitochondria to ER or plasma membrane Ca^2+^ channels, since their opening generates transient microdomains of high Ca^2+^ concentrations, a requisite feature for mitochondrial Ca^2+^ uptake due to low Ca^2+^ affinity of the mitochondrial Ca^2+^ uniporter (Spat et al., 2008). In particular, the mitochondrial Ca^2+^ uniporter complex mediates mitochondrial Ca^2+^ uptake following RyR activation in cardiac muscle fibrils (Szalai et al., 2000) and IP_3_R-mediated Ca^2+^ release in liver (Csordas et al., 2006). Intracellular Ca^2+^ channels also generate Ca^2+^ signals that affect the mitochondrial network in neurons, since the selective RyR agonist 4-chloro-m-cresol (4-CMC) induces mitochondrial fragmentation in neurons (SanMartín et al., 2012a), indicating that Ca^2+^ release from the ER has a pivotal role in shaping mitochondrial dynamics in hippocampal neurons.

Some oxidative and neurotoxic stressors increase mitochondrial fission (Rintoul et al., 2003; Barsoum et al., 2006; Pletjushkina et al., 2006). Persistent mitochondrial fission might impair mitochondrial function causing an increase in oxidative tonus, as observed in some neurodegenerative diseases. We have reported that exposure of primary hippocampal cultures to iron, which induces ROS generation and at high levels is neurotoxic, promoted mitochondrial fragmentation in most of the neurons present in the culture (SanMartín et al., 2014). We also reported that this fragmentation process requires functional RyR channels and that RyR-mediated mitochondrial Ca^2+^ uptake does not occur in fragmented mitochondria, probably due to impaired coupling of the mitochondrial Ca^2+^ uniporter with RyR channels (SanMartín et al., 2014). In addition, we found that pre-incubation of neurons with the antioxidant agent *N*-acetyl cysteine (NAC), a physiological precursor of cellular glutathione (GSH) synthesis, prevents the mitochondrial network fragmentation and RyR2 knockdown mediated by RyR channel activation in response to AβOs (SanMartín et al., 2012a; Lobos et al., 2016). These combined results corroborate the key role played by ROS and RyR on mitochondrial dynamics.

Of the three mammalian RyR isoforms, which are widely distributed in nervous tissues, the hippocampus expresses mainly the RyR2 isoform (Mori et al., 2000; Abu-Omar et al., 2017). In hippocampal neurons RyR2 is widely distributed in the soma, axon and dendritic tree (Hertle and Yeckel, 2007; Paula-Lima et al., 2011). Herein, we set out to investigate whether AβOs, at sub-lethal concentrations, induce redox sensitive RyR2-mediated mitochondrial Ca^2+^-uptake and ROS generation. We also investigated the possible protective effects of two antioxidant agents, NAC and the mitochondrial antioxidant agent EUK-134, against the negative impact of AβOs on mitochondrial function. The results presented here provide evidence that the neuronal dysfunction caused by acute AβOs treatment is driven at least in part by increased Ca^2+^ transfer from the ER to the mitochondria mediated by the RyR2 isoform, which is detrimental to Ca^2+^/ROS homeostasis in neurons.

## Materials and Methods

### Materials

Aβ peptide (Aβ_1-42_) was from Bachem Inc. (Torrance, CA, USA). Fluo4-AM, MitoSOX^TM^ Red Mitochondrial Superoxide Indicator, MitoTracker^®^ Orange CMTMRos, anti-rabbit Alexa Fluor^®^ 488 and anti-mouse Alexa Fluor^®^ 635 were from Molecular Probes, Inc. (Eugene, OR, USA). Hexafluoro-2-propanol (HFIP) and CMC were from Merck (Darmstadt, Germany), Neurobasal and Dulbecco’s modified essential medium (DMEM), B27 supplement and lipofectamine 2000 were from Gibco (Carlsbad, CA, USA). DOTAP Liposomal Transfection Reagent was from Sigma–Aldrich (Oakville, ON, Canada). Phosphodiester oligonucleotides (ODNs) were from Integrated DNA Technologies (Coralville, IA, USA). The mito-Pericam plasmid was donated by Dr. V. Eisner. Bicinchoninic acid assay (BCA) kit and mHsp-70 antibody were from Pierce Biotechnology (Rockford, IL, USA). Ryanodine was from Alexis (Lausen, Switzerland). PDVF membranes were from Millipore (Bedford, MA, USA). RyR2 antibody and Rhod2-AM was from Thermo-Fisher (Waltham, MA, USA). Gp91 ds-tat was from AnaSpec (Fremont, CA, USA).

### Preparation of AβOs

The Aβ_1-42_ peptide was prepared as previously described, as a HFIP film (De Felice et al., 2007; Paula-Lima et al., 2011; SanMartín et al., 2012a,b; Lobos et al., 2016). This film is dissolved next in DMSO to obtain a 5 mM stock solution, which is subsequently diluted with cold phosphate buffered saline (PBS) to 100 μM and incubated overnight at 4°C. After 24 h, the Aβ solutions (100 μM) were centrifuged at 4°C, 14,000 × *g* for 10 min to remove protofibrils and fibrils (insoluble aggregates). Supernatants with soluble AβOs were transferred to sterile tubes and protein levels were determined with a BCA kit. Fresh preparations of AβOs were used in all experiments.

### Primary Hippocampal Cultures

Eighteen-day-old embryos from Sprague-Dawley rats were used to obtain primary hippocampal cultures, as we previously described (Paula-Lima et al., 2005, 2011; SanMartín et al., 2012a,b, 2014, Lobos et al., 2016). Concisely, after meninges removal from brains, hippocampi were dissected and hippocampal cells were dissociated softly in HANKS-glucose solution. Cells were then centrifuged and resuspended in DMEM plus 10% horse serum and plated on polylysine-coated plates. After 1 h, DMEM was replaced by Neurobasal medium plus B-27. Cells were maintained for 15–21 days *in vitro* (DIV) in a humidified 5% CO_2_ atmosphere at 37°C prior to experimental handlings. Mature hippocampal cultures were enriched in neurons with a glial content <24% (Paula-Lima et al., 2011). This study was carried out in accordance with the recommendations of The Guidelines on the recognition of pain, distress and discomfort in experimental animals. The protocol was approved by the Bioethics Committee on Animal Research, Faculty of Medicine, University of Chile.

### Immunocytochemistry

Hippocampal cultures at 21 DIV were fixed by adding an equal volume of 4% formaldehyde and 4% sucrose (in PBS buffer) for 10 min, rinsed three times with PBS, incubated with 10% normal goat serum plus 0.1% Triton X-100 (blocking-permeant solution) for 1 h and then immunolabeled by overnight incubation at 4°C with mHsp-70 diluted in blocking solution (1/750). After this incubation period, cultures were rinsed three times with PBS and were incubated for 1 h at room temperature with Alexa Fluor^®^ 488 anti-rabbit as secondary antibody (1/400 in blocking solution). Cells were rinsed three times with PBS, and coverslips were mounted in DAKO mounting medium for morpho-topological analysis of the mitochondrial network. Quantification of the percentage of neurons with fragmented mitochondria was carried out as described previously (SanMartín et al., 2012a, 2014). To label mitochondria, cells were labeled for 20 min at 37°C with 50 nM MitoTracker Orange and observed on a Carl Zeiss LSM Pascal 5 confocal microscope system (Zeiss, Oberkochen, Germany) or on a Nikon C2+ confocal Microscope (Melville, NY, USA). Images were digitally acquired using LSM software (Zeiss) or NIS-Elements C software (Nikon). Image deconvolution and generation of zeta projections from 0.4 μm 7–15 stacks were performed using the ImageJ software program (National Institutes of Health, USA). Neurons were typed as exhibiting filamentous or fragmented mitochondrial network. Ten optical fields were observed for each condition, counting approximately 15 neurons. The percentage of neurons with fragmented mitochondria was determined respect to the total number of neurons counted.

### AβOs Treatment of Hippocampal Neurons

Neurons (14–21 DIV) were treated with 500 nM AβOs at the microscope stage, or for different incubation periods in the culture plates, depending on the type of experiment performed.

### Antisense Oligonucleotides

To down-regulate RyR2 expression, we used phosphodiester oligonucleotides (ODNs) with the following sequences. ODN RyR2: 5′-T^∗^T^∗^C GCCCGCATCAGCC^∗^A^∗^T-3′; ODN Scrambled (ODN Scr), 5′-C^∗^G^∗^GCAGGAGTCTGTG C^∗^G^∗^C-3. The ODN Scr was used as control, as previously described (Galeotti et al., 2008). Liposomal Transfection Reagent DOTAP (13 μM) was used to introduce ODNs into neurons. As controls, we also transfected neurons with ODNs specifically designed for the RyR1 and RyR3 isoforms; these ODNs did not modify RyR2 expression (data not shown).

### RyR2 Expression Levels after Oligonucleotide Transfection

RyR2 mRNA levels were determined by RT-PCR performed in a MX3000P Stratagene amplification system (La Jolla, CA, USA) using the DNA binding dye SYBR green and the following previously described Primer sense/Primer antisense sequences: 5′-AATCSanMartínGTGGCGGAATTTCTTG-3′/5′-TCTCCCTCAGCCTTCTCCGGTTC-3′ (Paula-Lima et al., 2011; Lobos et al., 2016). Levels of RyR2 mRNA were normalized respect to levels of β-actin mRNA and calculated by the relative 2-ΔΔCt method. For determination of RyR2 protein content, we performed western blot analysis. Cells homogenates were separated by SDS-PAGE (3.5–8% gradient or 10% polyacrylamide gels) and transferred to PVDF membranes for subsequent incubation with specific antibodies against RyR2 (Adasme et al., 2011; Paula-Lima et al., 2011).

### Determination of Intracellular Ca^2+^ Signals

Cells were preloaded with 5 μM Fluo4-AM in Tyrode solution (in mM: 30 glucose, 129 NaCl, 5 KCl, 2 CaCl_2_, 1 MgCl_2_, 25 HEPES-Tris, pH 7.3) for 30 min at 37°C. After washing three times with Tyrode, 500 nM AβOs were added to the cultures at the microscope stage and fluorescence images of intracellular Ca^2+^ signals were obtained every 15 s in an inverted confocal microscope (Carl Zeiss LSM Pascal 5) or every 3 s in an inverted confocal microscope (Nikon C2+). Regions of interest (ROIs) were determined in cell bodies and neurites. Relative Ca^2+^ levels are presented as *F*/*F*_0_ values, where *F*_0_ corresponds to the basal fluorescence and *F* to the experimental fluorescence. Experiments were done at room temperature (20–22°C).

### Transfection with the Mito-Pericam Plasmid and Determination of Mitochondrial Ca^2+^ Signals

Neurons at 14 or 15 DIV were transiently transfected with the mito-Pericam plasmid using a ratio of 1:3 DNA:lipofectamine 2000 as previously described (SanMartín et al., 2014). Twenty-four hours after transfection, cultures were treated with 50 μM ryanodine for 1 h, with 10 mM NAC for 30 min, or with vehicle. Next, cultures were washed three times with Tyrode solution and were maintained in this solution at the microscope stage. The 500 nM AβOs or 0.5 mM 4-CMC were added to the cultures. Mitochondrial Ca^2+^ signals from neuronal cells (identified as such by morphology) were recorded every 3 s in an Olympus Disk Scanning Unit (DSU) IX 81 confocal microscope (Olympus, Hamburg, Germany) using 60× oil immersion objective, excitation 420 nm and Hg/Ar lamp. Changes in Ca^2+^ levels are presented as *F*/*F*_0_ values, where *F* corresponds to the experimental fluorescence and *F*_0_ to the basal fluorescence. Experiments were done at room temperature (20–22°C).

### Simultaneous Measurements of Cytoplasmic and Mitochondrial Ca^2+^ Signals

Neurons at 14–21 DIV were incubated with 2.5 μM Rhod2 for 30 min, washed three times with Tyrode solution and incubated for additional 30 min to allow mitochondrial loading with Rhod2. Next, cells were transferred to Tyrode solution containing 5 μM Fluo4 and incubated for an additional 30 min period. Cells were then rinsed three times with Tyrode and AβOs (500 nM) were added to the cultures at the microscope stage. Simultaneous fluorescence images of intracellular and mitochondrial Ca^2+^ signals were obtained every 3 s in an inverted confocal microscope (Nikon C2+). ROIs were determined in cell bodies and neurites. Relative Ca^2+^ levels are presented as *F*/*F*_0_ values, where *F*_0_ corresponds to the basal fluorescence and *F* to the experimental fluorescence. Experiments were done at room temperature (20–22°C).

### Determination of Mitochondrial Superoxide Generation

Cultures were treated for 1 h with 50 μM ryanodine, for 30 min with 10 mM NAC, or for 2 h with 20 μM EUK-134, in Neurobasal medium supplemented with B-27. Cultures were then placed in modified Tyrode solution for subsequent loading with 1 μM MitoSOX for 20 min at 37°C. After washing three times with modified Tyrode solution, AβOs (500 nM) were added to the cultures at the microscope stage. The fluorescence images generated by the mitochondrial superoxide probe in primary hippocampal neurons (identified as such by morphology) were recorded every 5 s in a confocal microscope (Carl Zeiss LSM Pascal 5). Fluorescence signals are presented as *F*/*F*_0_ values, where *F*_0_ corresponds to the basal fluorescence levels and *F* to the experimental fluorescence. Experiments were performed at room temperature (20–22°C).

### Determination of Mitochondrial Hydrogen Peroxide Generation

Cultures at 14 or 15 DIV were transfected transiently with the HyperMito plasmid (Evrogen, Moscow, Russia) at a ratio of 1:3 DNA:lipofectamine 2000. Twenty-four hours after transfection, cultures were treated for 1 h with 50 μM ryanodine, for 30 min with 10 mM NAC, for 2 h with 20 μM EUK-134, or with vehicle in Neurobasal plus B27 medium. After three rinses with Tyrode solution, 500 nM AβOs were added to the cultures at the microscope stage. The fluorescent signals generated by the mitochondrial hydrogen peroxide probe were recorded from neuronal cells (identified as such by morphology) every 3 s in a confocal microscope (Carl Zeiss LSM Pascal 5). Relative mitochondrial hydrogen peroxide levels are presented as *F*/*F*_0_ values, where *F*_0_ corresponds to the basal fluorescence and *F* to the experimental fluorescence. Experiments were performed at room temperature (20–22°C).

### Morpho-topological Analysis

Mitochondria were identified by staining fixed cultures with mHsp-70, as we previously described (Paula-Lima et al., 2011; SanMartín et al., 2012a, 2014). The specificity of mHsp-70 as a mitochondrial stain was previously confirmed by staining mitochondria with MitoTracker Orange, which yielded the same labeling pattern as mHsp-70 (SanMartín et al., 2014). To determine the levels of the mitochondrial protein mHsp-70 in neurites and soma, segmentations were performed to define different ROIs, as described in detail elsewhere (SanMartín et al., 2014). Confocal image stacks were captured with a confocal microscope (Zeiss LSM-5, Pascal 5 Axiovert 200), using the LSM 5 3.2, and deconvoluted using Huygens Scripting (Scientific Volume Imaging, Hilversum, Netherlands).

### Determination of Mitochondrial Protein mHsp-70 in Soma and Neurites Volumes by 3D Reconstruction of the Segmented Objects

3D models were reconstructed from successive xy-images along the *z*-axis. Based on their volumes, we defined four different clusters to characterize mitochondrial connectivity as previously described (SanMartín et al., 2014). First, we determined the mean volume of single mitochondria, yielding 0.15 ± 0.04 μm^3^ (mean ± SE, *n* = 834). The mean volume of single mitochondria was used to define connected clusters: (i) 1–3 mitochondria (0–0.45 μm^3^); (ii) 4–10 mitochondria (0.45–1.5 μm^3^); (iii) 11–50 mitochondria (1.5–7.5 μm^3^); (iv) over 50 mitochondria (>7.5 μm^3^). Values obtained with control neurons were compared to those obtained from neurons treated with 500 nM or 1 μM AβOs for 24 h.

### Statistics

The significance of differences in the experiments was determined using paired Student’s *t*-test or one-way ANOVA followed by Bonferroni’s *post hoc* test.

## Results

### Inhibition of the NADPH Oxidase Type-2 Prevents the Emergence of AβOs-induced Cytoplasmic Ca^2+^ Signals

We have shown in previous work that AβOs generate Ca^2+^ entry signals via NMDA receptors, which promote RyR-mediated Ca^2+^-induced Ca^2+^ release (Paula-Lima et al., 2011). Stimulation of RyR channels by Ca^2+^ is redox sensitive and does not occur if RyR channel cysteine residues are highly reduced (Marengo et al., 1998). Accordingly, we tested if inhibition of the NADPH oxidase type-2 (NOX2), an important neuronal source of superoxide radical generation (Kishida and Klann, 2007; Ma et al., 2011; Riquelme et al., 2011), affected AβOs-induced cytoplasmic Ca^2+^ signals. As illustrated in **Supplementary Figure S1**, incubation of neurons with gp91-ds-tat, an inhibitory peptide of NOX2 activity that precludes its assembly (Rey et al., 2001), prevented the generation of Ca^2+^ signals in response to AβOs. In contrast, hippocampal cells incubated with a scrambled gp91-ds-tat peptide (scr), displayed similar Ca^2+^ signal generation in response to AβOs as controls. Based on these findings, we suggest that AβOs stimulate NOX2 activity, presumably via NMDA receptor stimulation (Brennan et al., 2009), and that the increased Ca^2+^ and ROS levels induced by AβOs jointly stimulate RyR-mediated Ca^2+^ release.

### Mitochondria Take Up Ca^2+^ Released via RyR Channels

**FIGURE 1:**
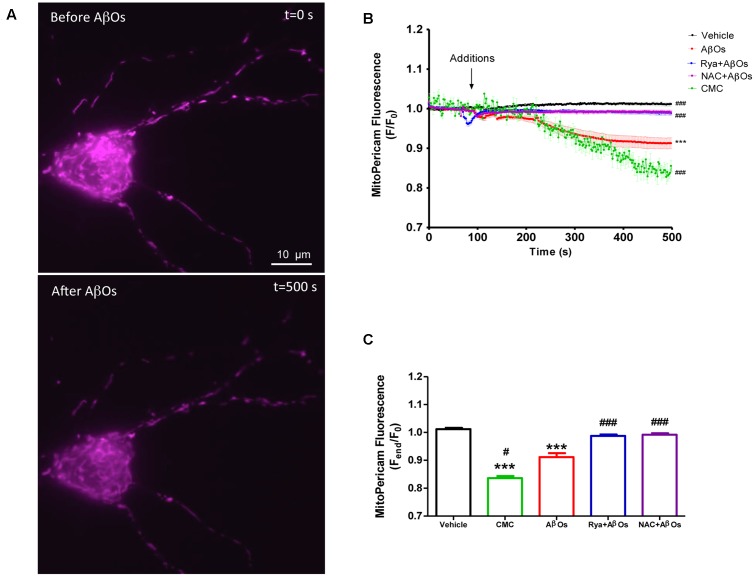
**Increased levels of mitochondrial Ca^2+^ induced by AβOs require the activation of RyR-mediated Ca^2+^ release.** Hippocampal neurons were transfected with a plasmid coding for the mito-Pericam protein specifically targeted to mitochondria, which decreases its fluorescence with increasing mitochondrial Ca^2+^ levels. **(A)** Representative images of mito-Pericam fluorescence responses recorded before and after addition of 500 nM AβOs. **(B)** Kinetics of mito-pericam fluorescence in neurons, before and after addition of 500 nM AβOs in the presence or absence of Ryanodine (Rya) 50 μM, pre-incubated for 1 h (to suppress RyR activity), or of the antioxidant NAC 10 mM, pre-incubated for 30 min. The arrow indicates the time of AβOs addition. Mitochondrial Ca^2+^ changes were also evaluated before and after the addition of the RyR agonist 4-CMC. Changes in fluorescence, plotted as the signal over time with respect to the baseline fluorescence (*F*/*F*_0_), are expressed as mean ± standard error. **(C)** Changes in fluorescence were plotted as the signal obtained at the end of the experiment (500 s) with respect to the baseline fluorescence (*F*_end_/*F*_0_), and were expressed as the mean ± standard error for all experimental conditions. In order to monitor Ca^2+^ levels at the mitochondria, for each condition regions of interest (ROIs) were defined in 1 to 3 neurons per field. (*n* = 17 for control, *n* = 24 for AβOs, *n* = 23 for AβOs + Rya, *n* = 9 for AβOs + NAC, *n* = 4 for CMC). Experiments were performed in triplicate, using at least three different cultures. Statistical analysis was performed using one-way ANOVA followed by Bonferroni *post hoc* test. ^∗∗∗^*p* < 0.001 compared with control. ^#^*p* < 0.05 and ^###^*p* < 0.001 compared to AβOs-treated neurons.

Mito-Pericam is a plasmid that expresses a Ca^2+^-sensing protein that decreases its fluorescence upon Ca^2+^ binding (Rizzuto et al., 1992), which is conjugated to a GFP derivative and a mitochondrial destination sequence. Hippocampal neurons transfected with mito-Pericam, represented by the false colored neuron illustrated in **Figure 1A**, were treated at the microscope stage with AβOs (500 nM) or with the RyR channel agonist 4-CMC (0.5 mM). Addition of AβOs (**Figure 1A**, bottom) produced a significant decrease in mito-Pericam fluorescence compared to that registered under basal levels (**Figure 1A**, top), indicating that AβOs induce mitochondrial Ca^2+^ entry. Quantification of fluorescence changes revealed that, within 1 min after AβOs or 4-CMC addition, neurons displayed significantly lower fluorescence relative to neurons treated with vehicle (**Figure 1B**); after 500 s, the decrease was significantly higher in 4-CMC-treated compared to AβOs-treated neurons.

To investigate whether RyR-mediated Ca^2+^ release from the ER underlies the AβOs-induced mitochondrial Ca^2+^ increase, we pre-incubated neurons for 1 h with 50 μM ryanodine (Rya), which in these conditions abolishes RyR-mediated Ca^2+^ release without causing Ca^2+^ depletion from the ER (Adasme et al., 2015). Interestingly, neurons pretreated with ryanodine did not display differences in mito-Pericam fluorescence after AβOs addition (**Figure 1B**). Previous work indicated that AβOs promote cytoplasmic ROS production (De Felice et al., 2007). Hence, we evaluated the participation of ROS in the mitochondrial Ca^2+^ increase induced by AβOs. To this aim, we pre-incubated neurons with 10 mM NAC for 30 min before the addition of AβOs. As illustrated in **Figure 1B**, NAC completely prevented the mitochondrial Ca^2+^ increase induced by AβOs. Quantification of the fluorescence recorded 500 s after AβOs addition, illustrated in **Figure 1C**, shows that both inhibitory ryanodine and NAC prevented AβOs-induced mitochondrial Ca^2+^ increase. Accordingly, we propose that AβOs induce Ca^2+^ uptake in mitochondria through RyR-mediated Ca^2+^ release, which requires in turn NMDA-receptor mediated Ca^2+^ entry and NOX2-mediated ROS generation.

### AβOs Induce RyR-Mediated Mitochondrial ROS Production

**FIGURE 2:**
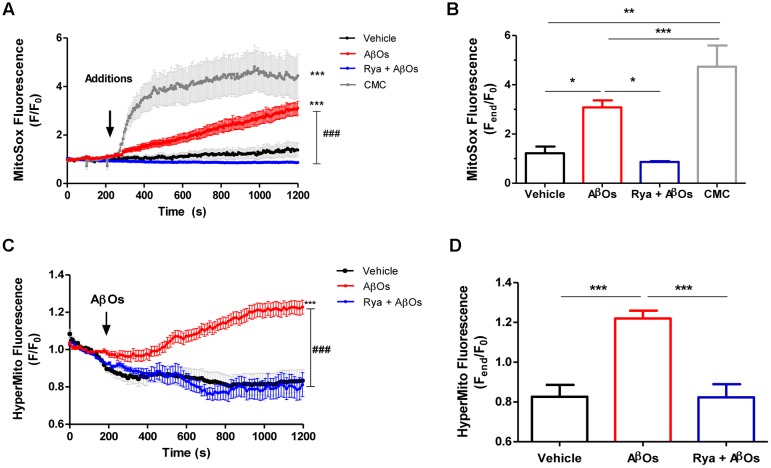
**The increased mitochondrial hydrogen peroxide and superoxide levels induced by AβOs require Ca^2+^ signals mediated by RyR.** Hippocampal neurons were loaded with MitoSOX^TM^, which is a fluorescent superoxide sensor, specifically targeted to mitochondria, or were transfected with the plasmid HyperMito, which encodes a fluorescent protein specifically targeted to mitochondria that acts as a hydrogen peroxide sensor. **(A)** Time course of MitoSOX^TM^ fluorescence responses recorded before and after addition of 500 nM AβOs to neurons pre-treated with Ryanodine 50 μM for 1 h (blue trace) or to untreated neurons (red trace). The arrow indicates the time of the addition of AβOs, or of the specific RyR agonist 4-CMC (1 mM, green trace), or vehicle (black trace). Changes in fluorescence, plotted as signal over time with respect to the baseline fluorescence (*F*/*F*_0_), were expressed as mean ± SE. **(B)** Changes in MitoSOX^TM^ fluorescence, plotted as the signal obtained at the end of the experiment (1200 s) with respect to the baseline fluorescence (*F*_end_/*F*_0_), were expressed as mean + SE for all the experimental conditions. (*n* = 6 for Control, *n* = 7 for AβOs, *n* = 6 for Rya + AβOs, *n* = 7 for CMC). **(C)** Time course of HyperMito fluorescence responses recorded before and after addition of 500 nM AβOs to neurons pre-incubated with Ryanodine 50 μM for 1 h (blue trace) or to control neurons (black trace). The arrow indicates the time of the addition of AβOs or vehicle. For each condition, ROIs were defined in 1 to 3 neurons per field in order to monitor the production of superoxide or hydrogen peroxide levels in the mitochondria. The experiments were repeated in triplicate using at least three different cultures (*n* ≥ 3). Changes in fluorescence, plotted as signal over time with respect to the baseline fluorescence (*F*/*F*_0_), were expressed as mean ± SE. **(D)** Changes in HyperMito fluorescence were plotted as the signal obtained at the end of the experiment (1200 s) with respect to the baseline fluorescence (*F*_end_/*F*_0_) and expressed as the mean + SE for all the experimental conditions. (*n* = 20 for Control, *n* = 32 for AβOs, *n* = 10 for Rya + AβOs). Statistical analysis was performed using one-way ANOVA followed by Bonferroni *post hoc* test. ^∗^*p* < 0.05, ^∗∗^*p* < 0.01, ^∗∗∗^*p* < 0.001 compared with control. ^###^*p* < 0.001 compared to AβOs-treated neurons.

To determine mitochondrial superoxide levels we used the MitoSOX^TM^ Red reagent (MitoSOX), comprised of a hydroethidine linked to a triphenylphosphonium cationic group that target this probe to the mitochondrial matrix in response to the negative membrane potential (Robinson et al., 2006). Oxidation of MitoSOX by superoxide produces red fluorescence signals. Stimulation of neurons with AβOs produced a rapid and sustained increase in MitoSOX fluorescence, indicating that AβOs promote mitochondrial superoxide generation; **Figure 2A** illustrates the time course of superoxide generation and **Figure 2B**, the fluorescence intensities obtained at the end of the experiment. Pre-incubation for 1 h with 50 uM ryanodine prevented the increase in probe fluorescence produced by AβOs, revealing that RyR-mediated Ca^2+^ release is essential to this process. In accord, neurons treated at the microscope stage with 4-CMC (0.5 mM), a RyR-channel agonist, exhibited an increase in MitoSOX fluorescence (**Figures 2A,B**), which was significantly higher than the increase produced by AβOs. The addition of vehicle did not change probe fluorescence.

To detect mitochondrial hydrogen peroxide generation, primary hippocampal cultures were transiently transfected with the Hyper^TM^-Mito plasmid. This plasmid codes for the mitochondrial protein HyPer-mito that has a circularly permuted yellow fluorescent protein inserted into the regulatory domain of the prokaryotic hydrogen peroxide-sensing protein (OxyR) (Belousov et al., 2006), allowing selective detection of mitochondrial hydrogen peroxide production in living cells. **Figure 2C** shows that addition of 500 nM AβOs produced within minutes a fluorescence increase in primary hippocampal neurons, indicating that AβOs promoted mitochondrial hydrogen peroxide generation (**Figure 2C**). In contrast, neurons in cultures pre-incubated for 1 h with 50 uM ryanodine to prevent RyR-mediated Ca^2+^ release did not exhibit changes in probe fluorescence in response to AβOs (**Figure 2C**). The quantification of the fluorescence recorded 1200 s after AβOs addition is shown in **Figure 2D**. Altogether, the combined results illustrated in **Figure 2** indicate that AβOs-induced RyR-mediated Ca^2+^ release has a key role in AβOs-induced mitochondrial superoxide and hydrogen peroxide generation.

### NAC and EUK-134 Prevent the Mitochondrial ROS Increase Induced by AβOs

**FIGURE 3:**
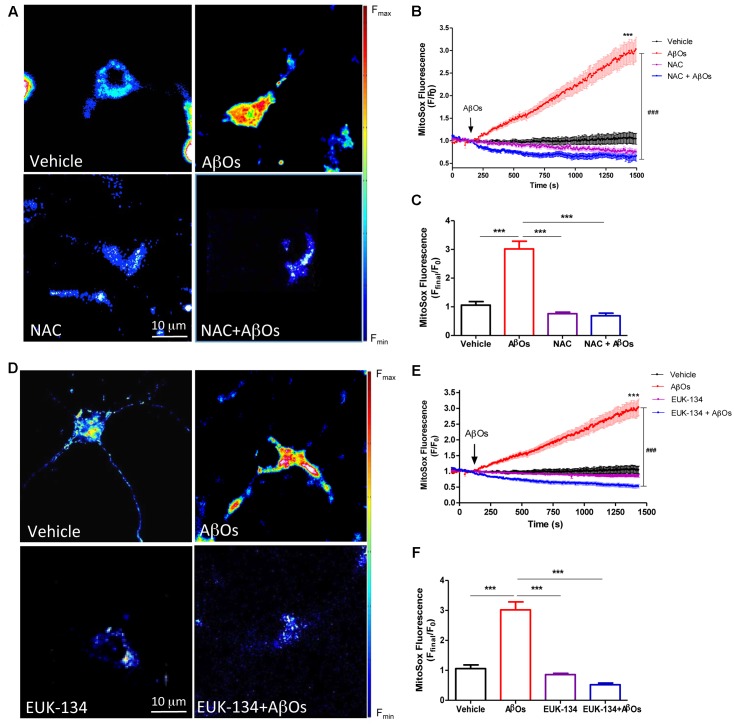
**The antioxidants NAC and EUK-134 prevent mitochondrial superoxide generation induced by AβOs.** **(A)** Hippocampal neurons, pre-incubated with 10 mM NAC for 30 min, were loaded next with MitoSOX^TM^ and treated with 500 nM AβOs or vehicle at the microscope stage. Pseudocolor images of MitoSOX^TM^ fluorescence in confocal sections obtained at the end of the experiment (1500 s). The scale bar corresponds to 10 μm. In the pseudo color rainbow scale (right), “warmer” colors correspond to higher fluorescence. **(B)** MitoSOX^TM^ fluorescence recorded as a function of time in neurons kept in vehicle (black trace), in neurons treated with AβOs (red trace), in neurons pre-incubated with NAC and kept in vehicle (pink trace) or treated with AβOs (blue trace). Changes in fluorescence, plotted as signal over time with respect to the baseline fluorescence (*F*/*F*_0_), were expressed as mean ± SE. Arrow indicates the time of AβOs additions. **(C)** Changes in MitoSOX^TM^ fluorescence were plotted as the signal obtained at the end of the experiment (1500 s) with respect to the baseline fluorescence (*F*_end_/*F*_0_) and were expressed as mean + SE for all experimental conditions. **(D)** Neurons were pre-incubated with 20 μM EUK-134 for 2 h, then loaded with MitoSOX^TM^ and treated with 500 nM AβOs or vehicle at the microscope stage. Pseudocolor images of MitoSOX^TM^ fluorescence were acquired as in **(A)**. **(E)** MitoSOX^TM^ fluorescence recorded as a function of time in neurons kept in vehicle (black trace), in neurons treated with AβOs (red trace), in neurons pre-incubated with EUK-134 and kept in vehicle (pink trace) or treated with AβOs (blue trace). Changes in fluorescence, plotted as signal over time with respect to the baseline fluorescence (*F*/*F*_0_), were expressed as mean ± SE. Arrow indicates the time of AβOs additions. **(F)** Changes in MitoSOX^TM^ fluorescence were plotted as the signal obtained at the end of the experiment (1500 s) with respect to the baseline fluorescence (*F*_end_/*F*_0_) and expressed as the mean + SE, for all the experimental conditions. For each condition, ROIs were defined in 1 to 3 neurons per field, in order to monitor the production of superoxide levels. (*n* = 21 for control, *n* = 31 for AβOs, *n* = 8 for NAC + AβOs, *n* = 12 for NAC), *n* = 16 for EUK-134 + AβOs, *n* = 20 for EUK-134). Statistical analysis was performed using one-way ANOVA followed by Bonferroni *post hoc* test. ^∗∗∗^*p* < 0.001 compared with control. ^###^*p* < 0.001 compared to AβOs-treated neurons.

We evaluated the effects of the general antioxidant NAC on the mitochondrial superoxide production induced by AβOs. For this purpose, neurons were pre-incubated for 1 h with NAC (10 mM), and then AβOs were added at the microscope stage. **Figure 3A** shows representative fluorescence images of mitochondrial superoxide generation recorded before (left) and 1500 s (right) after AβOs addition, in the presence or absence of NAC. As illustrated in these images, NAC prevented the mitochondrial superoxide increase elicited by AβOs. The quantification of the results from several experiments indicates that NAC completely prevented the superoxide increase in the mitochondria (**Figures 3B,C**). In agreement with these findings, cultures pre-incubated for 2 h with the mito-protector agent EUK-134 (20 μM) exhibited a significant decrease in neuronal superoxide levels following AβOs addition, as observed in the pseudo color images shown in **Figure 3D**. **Figure 3E** illustrates the quantification of the kinetics of the MitoSox fluorescence changes and **Figure 3F**, the endpoint fluorescence values.

**FIGURE 4:**
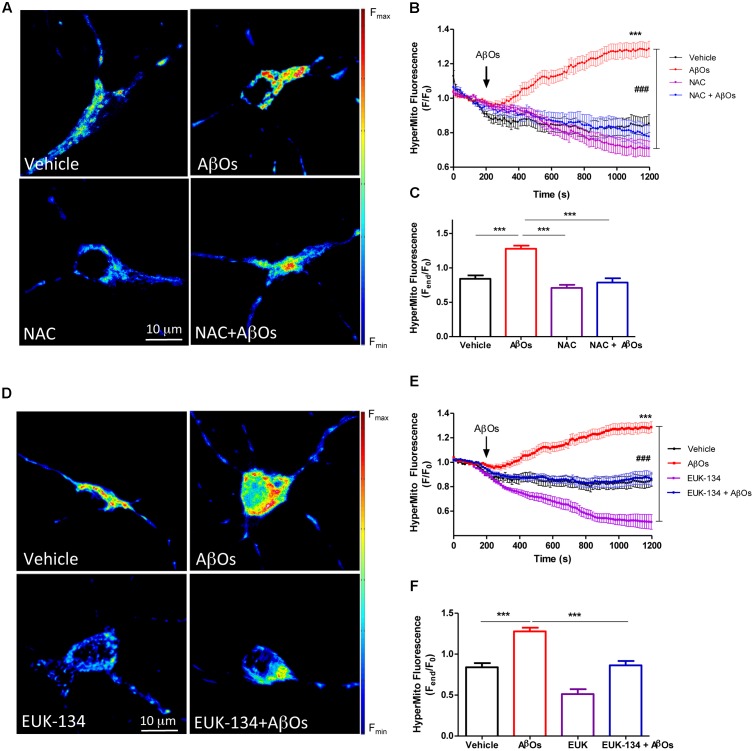
**The antioxidants NAC and EUK-134 prevent mitochondrial H_2_O_2_ generation induced by AβOs.** **(A)** Hippocampal neurons transfected with the plasmid HyperMito were pre-incubated with 10 mM NAC for 30 min and were then treated with 500 nM AβOs or kept in vehicle at the microscope stage. Pseudocolor images of HyperMito fluorescence in confocal sections obtained at the end of the experiment (1200 s). The scale bar corresponds to 10 μm. In the pseudo color rainbow scale (right), “warmer” colors correspond to higher fluorescence. **(B)** HyperMito fluorescence recorded as a function of time in neurons kept in vehicle (black trace), in neurons treated with AβOs (red trace), in neurons pre-incubated with EUK-134 and kept in vehicle (pink trace) or treated with AβOs (blue trace). Changes in fluorescence, plotted as signal over time with respect to the baseline fluorescence (*F*/*F*_0_), were expressed as mean ± SE. **(C)** Changes in HyperMito fluorescence were plotted as the signal obtained at the end of the experiment (1500 s) with respect to the baseline fluorescence (*F*_end_/*F*_0_) and expressed as the mean + SE, for all the experimental conditions. **(D)** Neurons were transfected with the plasmid HyperMito, pre-incubated with 20 μM EUK-134 for 2 h and treated with 500 nM AβOs or vehicle at the microscope stage. Pseudocolor images of HyperMito fluorescence obtained were obtained as in **(A)**. **(E)** HyperMito fluorescence recorded as a function of time in neurons kept in vehicle (black trace), in neurons treated with AβOs (red trace), in neurons pre-incubated with EUK-134 and kept in vehicle (pink trace) or treated with AβOs (blue trace). Changes in fluorescence were plotted as signal over time with respect to the baseline fluorescence (*F*_1_/*F*_0_) and expressed as mean ± SE. Arrow indicates the time of AβOs additions. **(F)** Changes in HyperMito fluorescence were plotted as the signal obtained at the end of the experiment (1500 s) with respect to the baseline fluorescence (*F*_end_/*F*_0_) and expressed as and expressed as the mean + SE, for all the experimental conditions. For each condition, ROIs were defined in 1 to 3 neurons per field in order to monitor the production of hydrogen peroxide levels. The experiments were repeated in triplicate using at least in three different cultures (*n* = 24 for Control, *n* = 36 for AβOs, *n* = 12 for NAC + AβOs, *n* = 12 for NAC, *n* = 16 for EUK-134 + AβOs, *n* = 32 for EUK-134). Statistical analysis was performed using one-way ANOVA followed by Bonferroni *post hoc* test. ^∗∗∗^*p* < 0.001 compared with control. ^###^*p* < 0.001 compared to AβOs.

The effects of NAC and EUK-134 on AβOs-induced mitochondrial hydrogen peroxide production were tested next. For this purpose, cultures were pre-incubated with NAC and EUK-134 as described above. **Figure 4A** shows representative fluorescence images of neuronal mitochondrial H_2_O_2_ generation before (left) and 1200 s (right) after AβOs addition, in the presence or absence of NAC (**Figure 4A**) or EUK-134 (**Figure 4D**). Both NAC and EUK-134 prevented the increase in mitochondrial H_2_O_2_ levels caused by AβOs addition. Quantification of the kinetics of hydrogen peroxide generation is shown in **Figures 4B,E**, while the fluorescence intensities measured at the endpoint of the experiments are shown in **Figures 4C,F**.

Based on these combined findings, we conclude that both antioxidants, NAC and EUK-134, prevent the increases in mitochondrial superoxide and hydrogen peroxide levels induced by AβOs.

### AβOs Induce Mitochondrial Fragmentation in Hippocampal Neurons and EUK-134 Prevents This Effect

**FIGURE 5:**
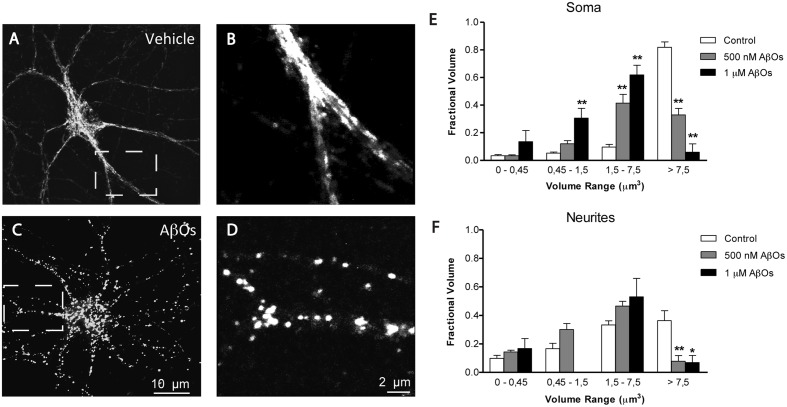
**AβOs induce dose dependent fragmentation of mitochondrial network.** **(A)** Representative image of Hsp-70 immunofluorescence (green) used as a marker of the mitochondrial network in a control neuron. **(B)** Amplification of the white box in **(A)**. **(C)** Representative image of Hsp-70 immunofluorescence in a neuron from a culture treated with AβOs for 24 h. **(D)** Amplification of the white box in **(C)**. Analysis of the mitochondrial clusters in soma **(E)** and neurites **(F)**; empty bars correspond to control neurons and black bars, to neurons treated with AβOs. The calibration bar in **(C)** corresponds to 10 μm, and in **(D)**, to 2 μm. Values represent mean + SE (*n* = 7 cells analyzed per condition from three independent cultures). Statistical significance was analyzed by one-way ANOVA followed by Bonferroni’s *post hoc* test. ^∗^*p* < 0.05; ^∗∗^*p* < 0.01 compared to controls.

We described previously that AβOs promote the fragmentation of the mitochondrial network, and that the antioxidant NAC prevents AβOs-induced mitochondrial fragmentation by preventing RyR-mediated Ca^2+^ release (SanMartín et al., 2012a). To quantify independently changes in mitochondrial network in the soma and neurites, we performed a detailed morpho-topological analysis of the mitochondrial network before and after the exposure to 500 nM AβOs. This analysis defined four mitochondrial clusters according to their volume. The mean volume of single mitochondria (0.15 ± 0.04 μm^3^) was used to define all clusters (see Materials and Methods). As previously described (SanMartín et al., 2014), the mitochondrial network of hippocampal neurons in control conditions is highly interconnected, with elongated mitochondria that extend across the cell body and neuronal projections. **Figure 5A** illustrates mature hippocampal neurons (18–21 DIV) displaying a characteristic organization of their mitochondrial network, which may reflect specific cellular demands in the neuronal soma (**Figure 5A**) and neurites (**Figure 5B**). Compared to a representative control neuron (**Figures 5A,B**), the continuity of the mitochondrial network of a neuron incubated for 24 h with 500 nM AβOs exhibited a loss, and the proportion of small mitochondria in soma and neurites increased (**Figures 5C,D**). The quantification of morpho-topological analysis of mitochondrial fragmentation revealed that AβOs decreased the fraction of the biggest clusters (>7.5 μm^3^) and increased the proportion of the intermediate clusters of mitochondria (1.5–7.5 μm^3^), in the soma (**Figure 5E**) as well as in the neurites (**Figure 5F**). This effect was dose-dependent, but we set the subsequent experiments with the lower concentration of AβOs, 500 nM, which we have reported to be sub-lethal (Paula-Lima et al., 2011).

**FIGURE 6:**
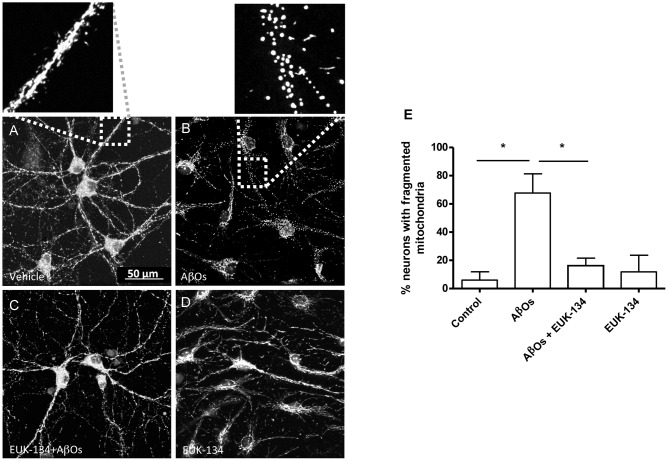
**The antioxidant EUK-134 prevents the mitochondrial network fragmentation induced by AβOs.** **(A–D)** Fluorescence confocal images of neurons labeled with 0.05 μM MitoTracker Orange for 15 min and fixed as detailed in the text. **(A)** Control conditions. **(B)** Images collected from neuronal cultures after incubation with AβOs (500 nM, 24 h). The insets show the amplification of the white boxes in **(A,B).** **(C)** Images collected from neuronal cultures after pre-incubation with EUK-134 and subsequent incubation with AβOs. **(D)** Images collected from neuronal cultures after pre-incubation with EUK-134 alone. **(E)** Quantification of the fraction of neurons exhibiting fragmented mitochondrial networks. Data are given as mean + SE. (*n* = 3, with 3–10 neurons counted per confocal field; 4 confocal fields were analyzed for each experimental condition in three different cultures). Statistical significance was analyzed by one-way ANOVA followed by Bonferroni’s *post hoc* test. ^∗^*p* < 0.05.

We investigated next the effects of the mitochondrial antioxidant EUK-134 on the mitochondrial fragmentation induced by AβOs. Analysis of fixed control neurons stained with MitoTracker Orange revealed that only 5% of primary hippocampal neurons contained fragmented mitochondria, while most of the neurons exhibited filamentous mitochondria in neurites and soma (**Figure 6A**). In contrast, a significantly higher percentage of neurons (67%) treated with 500 nM AβOs for 24 h contained punctuate mitochondria, exposing noteworthy fragmentation of the mitochondrial network (**Figure 6B**). Incubation with 20 μM EUK-134 before AβOs treatment significantly decreased (from 67 to 16%, **Figure 6C**) the fraction of neurons exhibiting fragmented mitochondria; 20 μM EUK-134 by itself (**Figure 6D**) did not elicit significant changes in the content of fragmented mitochondrial (11%) when compared to the controls (**Figure 6E**). Theses results indicate that the EUK-134 mitochondrial antioxidant prevents mitochondrial fragmentation induced by AβOs.

### The RyR2 Isoform Plays a Key Role in AβOs-induced Mitochondrial Ca^2+^ Overload and Fragmentation

Oligotransfection of primary hippocampal cultures with an oligodeoxynucleotide against RyR2 (ODN RyR2) reduced by 50% RyR2 mRNA (**Supplementary Figure S2A**) and protein contents (**Supplementary Figure S2B**), determined in homogenates of the whole primary culture. We studied next the impact of RyR2 knockdown on agonist-induced RyR-mediated cytoplasmic Ca^2+^ signals elicited by 4-CMC, and found that neurons in ODN RyR2 transfected cultures exhibited 40% lower Ca^2+^ signals when compared to neurons present in cultures transfected with the scrambled oligonucleotide (ODN Scr) (**Supplementary Figure S2C**).

**FIGURE 7:**
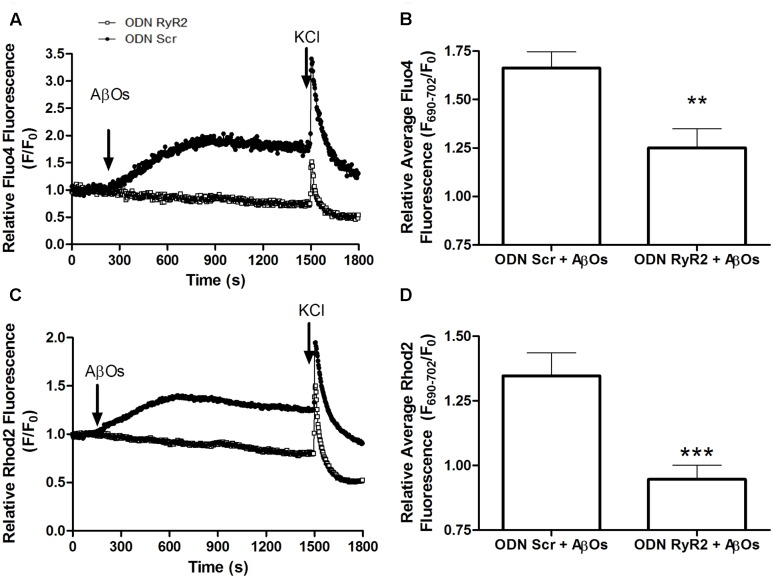
**RyR2 is required for the cytoplasmic and mitochondrial Ca^2+^ increases induced by AβOs.** Fluorescence confocal images of neurons labeled with 1 μM Rhod2 plus 5 μM Fluo-4 as described in detail in the text. **(A)** Changes in Fluo4 fluorescence, determined in ODN Scr and ODN RyR2 transfected neurons, were normalized with respect to the baseline fluorescence (*F*/*F*_0_) in a representative experiment. The arrows indicate the time of addition of 500 nM AβOs and 50 mM KCl. **(B)** Quantification of the average changes in Fluo4 fluorescence observed in neurons transfected with ODN Scr or ODN RyR2; 12 min after AβOs addition values were recorded for 15 s and expressed as *F*/*F*_0_ (mean + SE). For each condition, ROIs were defined in at least four neurons per field in order to monitor the Ca^2+^ levels in the cytoplasm. The experiments were repeated in triplicate using three different cultures (*n* = 12 cells per condition). Statistical analysis was performed using two-tailed unpaired *t*-test; ^∗∗^*p* < 0.01. **(C**) Changes in Rhod2 fluorescence in ODN Scr and ODN RyR2 transfected neurons were plotted as the signal normalized with respect to the baseline fluorescence (*F*/*F*_0_); the figure shows a representative experiment. The arrows indicate the times of 500 nM AβOs and 50 mM KCl addition. **(D)** Quantification of the average changes in Rhod2 fluorescence observed in ODN Scr and ODN RyR2 transfected neurons, recorded and analyzed as in **(B)**. ^∗∗∗^*p* < 0.001.

To evaluate if RyR2 knockdown affected AβOs-induced cytoplasmic and mitochondrial Ca^2+^ signals, we used neuronal cultures transfected with ODN RyR2 or ODN Scr and loaded with Fluo4 and Rhod2 (for a representative experiment, see **Supplementary Figure S3**). As reported previously (Sanz-Blasco et al., 2008; Paula-Lima et al., 2011; SanMartín et al., 2012a; Hedskog et al., 2013), we confirmed that treatment with AβOs caused an increase in both cytoplasmic and mitochondrial Ca^2+^ signals. The fluorescence of both dyes increased in response to 50 mM KCl addition at the end of the experiment, evidencing that neurons were still active after all the experimental manipulations (**Supplementary Figure S3**). The fluorescence intensities observed in ODN RyR2 and ODN Scr-treated neurons revealed that ODN RyR2 transfection caused a significant reduction in both the cytoplasmic (**Figure 7A**) and the mitochondrial (**Figure 7B**) Ca^2+^ signals induced by AβOs. The quantification of the last fifteen seconds of the average of three experiments shows that these differences are statistically significant (**Figures 7C,D**).

**FIGURE 8:**
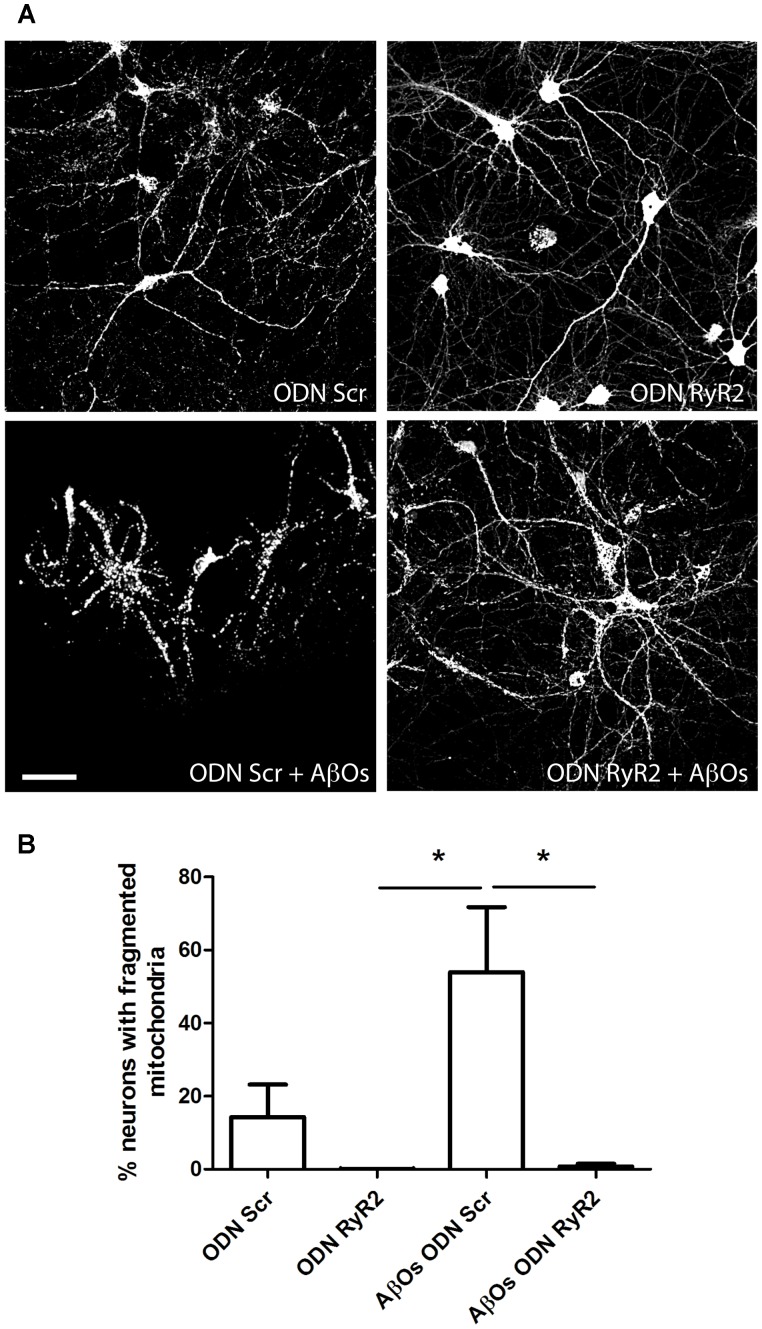
**Ca^+2^ release mediated by RyR2 promotes the mitochondrial fragmentation induced by AβOs.** **(A)** Fluorescence confocal images were recorded in neurons transfected with ODN Scr and ODN RyR2, and incubated with 0.05 μM MitoTracker Orange for 15 min. Images collected from neuronal cultures after incubation with AβOs (500 nM, 24 h) or vehicle. The calibration bar in **(A)** corresponds to 50 μm. **(B)** Quantification of the fraction of neurons exhibiting fragmented mitochondrial networks. Data represent mean + SE (*n* = 4 experiments with different cultures, with 3–10 neurons counted per confocal field analyzed; 3–4 confocal fields were analyzed for each experimental condition). Statistical significance was analyzed by one-way ANOVA followed by Bonferroni’s *post hoc* test. ^∗^*p* < 0.05.

We further investigated the effects of transfection with ODN RyR2 on AβOs-induced mitochondrial fragmentation. Analysis of control neurons transfected with ODN Scr and loaded with MitoTracker Orange, revealed that about 15% of primary hippocampal neurons contained fragmented mitochondria, whereas 24 h after 500 nM AβOs addition 53% exhibited punctuate mitochondria, revealing fragmentation of the mitochondrial network (**Figures 8A,B**). Transfection with ODN RyR2 markedly reduced almost to zero the percentage of neurons that exhibited a mitochondrial punctuate pattern, even after AβOs treatment (**Figures 8A,B**). Based on these results, we propose that Ca^2+^ release mediated by the RyR2 isoform plays a central role in AβOs-induced mitochondrial fragmentation.

## Discussion

The concentration of AβOs in cerebral cortex brain tissue isolated from controls or from AD patients varies from 50 nM to 2 μM (Yang et al., 2017). We used the sub-lethal AβOs concentration of 500 nM, which is deleterious to neuronal function because it inhibits long term potentiation (Wang et al., 2002; Schlenzig et al., 2012) and produces aberrations in synapse composition, shape and density (Lacor et al., 2007). Furthermore, treatment with 500 nM AβOs increases reactive oxygen species (ROS) levels (De Felice et al., 2007; Lobos et al., 2016), decreases non-transferrin-bound iron uptake (SanMartín et al., 2012b) and induces differential gene expression (Sebollela et al., 2012). Addition of 500 nM AβOs increases cytoplasmic calcium in primary hippocampal (Paula-Lima et al., 2011) and cortical neurons (Ferreira et al., 2014) and cerebellar granule cells (Sanz-Blasco et al., 2008), and results in depolarization of mitochondrial membrane potential in primary cortical neurons (Ferreira et al., 2014) and cerebellar granule cells (Sanz-Blasco et al., 2008), among other effects. Moreover and closely related to our present results, 800 nM AβOs induce loss of dendritic spines and promote mitochondrial fission in rat hippocampal primary cultures (Wang et al., 2009).

We reported that AβOs increase intracellular Ca^2+^ signals in primary hippocampal neurons by promoting Ca^2+^ entry through NMDA receptors; this increase does not occur in neurons pre-incubated with inhibitory ryanodine, showing that RyR-mediated Ca^2+^ release is required for the cytoplasmic Ca^2+^ increase induced by AβOs (Paula-Lima et al., 2011). RyR protein isoforms have highly reactive cysteine residues, a property that led to the proposal that RyR channels act as intracellular redox sensors (Hidalgo, 2005). Furthermore, RyR channel activation by Ca^2+^ does not occur if these cysteine residues are in the reduced state (Marengo et al., 1998). Consistent with the dependence of RyR-mediated Ca^2+^ release on neuronal redox state (Bull et al., 2008), we have reported that pre-incubation with the general antioxidant NAC inhibits AβOs-induced cytoplasmic Ca^2+^ signal generation (SanMartín et al., 2012a). Here, we add to these previous reports by showing that NOX2 inhibition significantly prevented the cytoplasmic Ca^2+^ signals induced by AβOs. Thus, the present findings further support our previous proposal that RyR-mediated Ca^2+^ release induced by AβOs requires AβOs-induced ROS generation to increase the activity of RyR channels (Paula-Lima et al., 2011).

**FIGURE 9:**
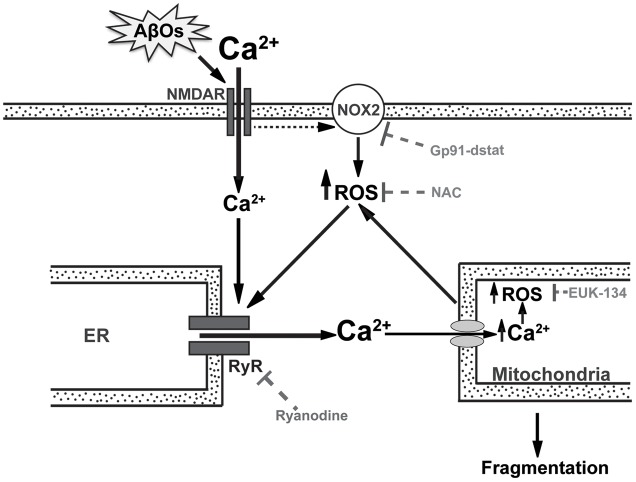
**RyR-mediated Ca^2+^ release is a key component in the mitochondrial Ca^2+^ and ROS increases and the mitochondrial fragmentation induced by AβOs.** In the post-synaptic compartment, AβOs induce Ca^2+^ entry through NMDA receptors (Paula-Lima et al., 2011) and stimulate NOX2 activity, presumably via NMDA receptor stimulation (Brennan et al., 2009). Endoplasmic reticulum (ER) resident RyR channels are redox sensitive and their activation by Ca^2+^ does not occur if RyR channel cysteine residues are highly reduced (Marengo et al., 1998). Thus, the NMDA-dependent increases in Ca^2+^ and ROS levels induced by AβOs would jointly stimulate redox-sensitive RyR-mediated Ca^2+^-induced Ca^2+^ release (CICR) from the ER, amplifying the Ca^2+^ signal initiated by Ca^2+^ influx through NMDA receptors. As a result, mitochondria take up Ca^2+^ via the mitochondrial channel uniporter or the Letm1/H+ antiporter (Finkel, 2015), which promotes mitochondrial ROS production, which in turn oxidize and activate more RyR channels in a vicious feedback cycle. Incubation of neurons with gp91-ds-tat, an inhibitory peptide of NOX2 activity, prevents the generation of Ca^2+^ signals in response to AβOs. Ryanodine and NAC prevent the mitochondrial Ca^2+^ and ROS increase as well as the mitochondrial fragmentation caused by AβOs (Paula-Lima et al., 2011; SanMartín et al., 2012a). EUK-134, which is a mito-protector antioxidant, also prevents the ROS increase and the mitochondrial fragmentation induced by AβOs. This scheme supports the idea that anomalous RyR-mediated Ca^2+^ release is a key component in the mitochondrial Ca^2+^ and ROS increase and the fragmentation of mitochondria induced by AβOs in hippocampal neurons. The present results show conclusively that the general antioxidant NAC and the mitochondrial protective agent EUK-134 significantly prevent AβOs-induced mitochondrial dysfunctions.

Due to the activity of the electron transport chain, mitochondria are the major sources of superoxide and hydrogen peroxide production in cells even under physiological conditions (Mari et al., 2009). An increase in ROS production and oxidative damage is a characteristic feature of AD and other neurodegenerative pathologies, such as Parkinson’s disease (Peng and Jou, 2010; Marchesi, 2011; Yan et al., 2013). These findings raise the possibility that the neuronal damage produced by AβOs may be due at least in part to excessive ROS generation. In fact, the results presented in this work show that AβOs-induced ROS generation causes anomalous RyR-mediated Ca^2+^ signals, which by promoting Ca^2+^ entry into the mitochondria generate even more ROS and thus create a noxious positive feedback cycle (**Figure 9**).

A previous report showed that neurons treated for 1 h with 500 nM AβOs display increased mitochondrial superoxide generation, measured with the MitoSOX probe (Ma et al., 2011). Here, we added to these findings by showing the fast kinetics of AβOs-induced mitochondrial ROS production. Thus, addition of 500 nM AβOs to hippocampal neurons increased mitochondrial superoxide levels as early as within 15 s and increased hydrogen peroxide levels within 50 s. The rate of increase in the levels of these two ROS species remained constant over time, reaching significant differences compared to control conditions. These results support the proposal that, in response to AβOs, mitochondria generate superoxide anion radicals that undergo fast dismutation to hydrogen peroxide, which in turn diffuses to the cytoplasm through the mitochondrial membrane. Here, we also report that pre-incubation with the antioxidant NAC or with the mitochondrial mito-protector EUK-134, prevented the mitochondrial increments in superoxide and hydrogen peroxide levels produced by AβOs. However, it is worth noting that both the MitoSOX and the HyperMito probes display some limitations in detecting mitochondrial ROS (Roma et al., 2012; Dikalov and Harrison, 2014).

Uncontrolled mitochondrial ROS generation may interfere with the morphology of the mitochondrial structure. The energetic requirements of a cell are related to its function and to the number of mitochondria, their morphology and distribution in the cytoplasm, which is particular to each type of cell (Kuznetsov et al., 2009). In the polarized neuronal morphology, mitochondrial distribution and structure have to fulfill the ATP requirements of the axon and dendrites (Knott et al., 2008). Mitochondria form a vastly interconnected network in the soma of neurons, with predominant large filamentous structures. In neurites, this network is more disordered, showing different structures and sizes of mitochondria. Previously, we described different mitochondrial structures and sizes in soma and neurites in control hippocampal neurons (SanMartín et al., 2014). We reported also that the presence of a putative neurotoxic agent such as iron, which induces ROS generation, promotes mitochondrial fission in soma and neurites (SanMartín et al., 2014).

The first evidence linking AD with modifications in the structure of the mitochondrial network was reported in fibroblasts from AD patients, which exhibit increased fused mitochondria presumably caused by a decrease in the expression of the fission protein Drp-1 (Wang et al., 2008a). Subsequent studies, (Wang et al., 2008b) showed that overexpression of the APP protein in a neuroblastoma cell line induces mitochondrial fragmentation, probably due to increased Aβ peptide production. Furthermore, incubation of hippocampal neurons in culture with AβOs induces loss of dendritic spines and mitochondrial fission (Wang et al., 2009). Despite evidence showing that mitochondrial fission occurs in cellular models of AD, the role of ROS in this process remains undefined. Previous studies addressed the effects of ROS on mitochondrial dynamics in cerebellar granule neurons, in which hydrogen peroxide produces fragmentation of the mitochondrial network prior to cell death by apoptosis (Jahani-Asl et al., 2007); yet, these authors did not investigate further the mechanisms leading to mitochondrial fission.

We have reported that the proportion of hippocampal neurons with punctuate mitochondrial morphology increases following treatment with AβOs (Paula-Lima et al., 2011; SanMartín et al., 2012a). This increase does not occur in neurons pre-incubated with inhibitory concentrations of ryanodine or the antioxidant NAC, both of which prevent Drp-1 translocation to the mitochondria (Paula-Lima et al., 2011; SanMartín et al., 2012a). Given the above, we proposed that NAC acts at the level of RyR, reducing highly reactive RyR cysteines and thus preventing RyR-mediated Ca^2+^ release from the ER. As a result, mitochondria would fail to take up Ca^2+^, preventing the increased ROS production caused by Ca^2+^ uptake. Previous reports indicate that NAC protects the hippocampus from oxidative stress, apoptosis, and Ca^2+^ entry (Naziroglu et al., 2014); NAC also modulates inflammation and prevents cognitive and memory damage in traumatic brain injury induced in rats (Haber et al., 2013). Furthermore, a proteomic study of brain proteins in a transgenic model of AD (human double mutant knock-in mice APP/PS-1) supports the idea that NAC may be beneficial *in vivo* for increasing cellular stress responses and for influencing the levels of energy- and mitochondria-related proteins (Robinson et al., 2011). In accord, NAC treatment prevents brain oxidative stress in the same transgenic model (Huang et al., 2010) and against memory deficits in mice intracerebroventricularly injected with amyloid beta-peptide (Fu et al., 2006). Oral supplementation with NAC also reverses the abnormalities in long-term potentiation observed in aged animals (Robillard et al., 2011). Furthermore, the use of NAC in bipolar disorder and schizophrenia may possess therapeutic potential in the field of psychiatric research (Dean et al., 2015; Oliver et al., 2015).

We report here that treatment of hippocampal neurons with AβOs (500 nM or 1 μM) for 24 h, increased the population of mitochondria with volumes <7.5 μm^3^ both in soma and neurites. This change in neuronal mitochondrial structure increased in a dose dependent manner. Moreover, we found that pre-incubation of primary hippocampal cultures with EUK-134 reduced the number of neurons displaying fragmented mitochondria. Hence, we propose that increased mitochondrial ROS levels play an important role in the mitochondrial fragmentation induced by AβOs. Of note, increases in the basal levels of cytoplasmic Ca^2+^, abnormal Ca^2+^ signals, increased ROS levels and increased punctuate mitochondrial phenotype are hallmarks of the AD pathology. Based on our results, we propose that anomalous RyR-mediated Ca^2+^ release is a key component in the mitochondrial Ca^2+^ and ROS increase and the fragmentation of mitochondria induced by AβOs in hippocampal neurons. Moreover, our combined findings show conclusively that the general antioxidant NAC and the mitochondrial protective agent EUK-134 significantly prevent AβOs-induced mitochondrial dysfunctions.

## Conclusion

We describe here novel findings highlighting the key role of the RyR2 isoform in the mitochondrial dysfunctions induced by acute AβOs treatment. We showed previously that RyR2 up-regulation accompanies the increase in spine density induced by BDNF; RyR2 up-regulation also occurs following high frequency field stimulation of primary hippocampal cultures and spatial memory training (Zhao et al., 2000; Adasme et al., 2011; Riquelme et al., 2011). Conversely, treatment with AβOs for 1–6 h causes a decrease in RyR2 protein levels in primary hippocampal neurons (Paula-Lima et al., 2014), as does AD in its initial stages (Kelliher et al., 1999). We show in this work that RyR2 knockdown suppresses the Ca^2+^ transfer from the ER to the mitochondria induced by acute treatment with AβOs, and prevents the ensuing disruption of the mitochondrial network. Based on these results, we propose that the initial RyR2 down-regulation induced by AβOs represents an early protective neuronal response from the RyR2-mediated noxious effects of AβOs on mitochondrial function, which presumably contribute to AβOs-induced early synaptotoxicity. This proposal agrees with previous findings showing that ryanodine, at inhibitory concentrations, prevents the mitochondrial fragmentation induced by acute AβOs treatment (Paula-Lima et al., 2011). In addition, AβOs-induced early RyR2 down-regulation is likely to prevent the increase in dendritic spine density induced by hippocampal neuronal activity; this impairment would further the initial synaptic dysfunctions induced by AβOs. Nonetheless, longer incubations (24 h) with AβOs restore RyR2 protein content to control levels (Paula-Lima et al., 2011). Therefore, we propose that hippocampal neuronal cells fail to sustain this early response over time, and that the delayed recovery of RyR2 levels, by causing mitochondrial dysfunction, contributes to AβOs-induced neuronal injury.

## Author Contributions

CS and PV performed most of the experimental work and analysis. TA contributed with the experimental design, performed some of the experiments and generated the final scheme presented as **Figure 9**. PL performed the analysis of some experiments, and contributed to the writing of the manuscript. BB was the responsible for Abeta oligomers preparations. JG was in charge of the primary hippocampal cultures. AG and SH provided support for microscopy image analysis. CH and AP-L participated in the experimental design, in the interpretation of the results, in manuscript writing and also provided most of the financial support for the work.

## Conflict of Interest Statement

The authors declare that the research was conducted in the absence of any commercial or financial relationships that could be construed as a potential conflict of interest.

## Supplementary Material

**FIGURE S1 | NOX2 inhibition prevents the emergence of AβOs-induced cytoplasmic Ca^2+^ signals.** Hippocampal cells preloaded for 30 min at 37°C with 5 mM Fluo4-AM were treated with AβOs (500 nM) at the microscope stage. **(A)**. Fluorescence signals were recorded during 1200 s (red trace). The blue trace represents the fluorescence collected from neurons pre-incubated for 30 min with 1 mM gp91-ds-tat to inhibit NOX2 activity; these neuronal cells did not generate Ca^2+^ signals in response to AβOs. In contrast, hippocampal neurons incubated with a scrambled inactive peptide of gp9-ds1-tat (scr) presented similar Ca^2+^ signal generation in response to AβOs (green trace) as controls. Changes in fluorescence were plotted as *F*/*F*_0_, and were expressed as the mean ± standard error. Quantification of the signals recorded at the end of the record (1200 s) is shown in **(B)**. Data are given as mean ± SE. (*n* = 8 for AβOs, *n* = 6 for gp91-ds-tat + AβOs, *n* = 5 for gp91 Scr + AβOs), Statistical significance was analyzed by one-way ANOVA followed by Bonferroni’s *post hoc* test. ^∗∗^*p* < 0.01 compared to AβOs; ^###^*p* < 0.001 compared to gp91-ds-tat + AβOs.

**FIGURE S2 | Antisense oligonucleotides decrease RyR2 expression.** Neurons were transfected with antisense oligonucleotides against RyR2 (ODN RyR2) or with a scrambled sequence as a control (ODN Scr), using Lipossomal DOTAP transfection reagent for 24 h. **(A)** Determination of mRNA by qPCR assay. (*n* = 3). **(B)** Determination of RyR2 protein levels by Western blot analysis. A representative blot is shown in the inset. Results are expressed as mean + SE (*n* = 4). **(C)** ODN RyR2 (open squares) and ODN Scr (filled circles) transfected neurons were preloaded for 30 min at 37°C with 5 mM Fluo4-AM and treated with 4-CMC (1 mM) at the microscope stage. Fluorescence signals were recorded during 1200 s and changes in fluorescence were plotted as *F*/*F*_0._ Data are given as mean ± SE. (*n* = 13 for Oligo RyR2 and 7 for Oligo Scr). Statistical significance was analyzed by two-tailed unpaired *t*-test. ^∗^*p* < 0.05.

**FIGURE S3 | Fluo4 and Rhod2 fluorescence signals induced by AβOs in hippocampal neurons.** Representative experiment of time-dependent florescence changes recorded in neurons transfected with ODN Scr and loaded with 1 μM Rhod2 and 5 μM Fluo-4 as detailed in the text. At the microscope stage, neurons were treated with 500 nM AβOs and Fluo4 and Rhod2 fluorescence signals were recorded in two separate channels, which did not overlap.
