# Supplementary figures and images for: RyR2-Mediated Ca2+ Release and Mitochondrial ROS Generation Partake in the Synaptic Dysfunction Caused by Amyloid β Peptide Oligomers
Source: Front Mol Neurosci. 2017 Apr 25;10:115. doi: 10.3389/fnmol.2017.00115 (PMC5403897; doi:10.3389/fnmol.2017.00115)

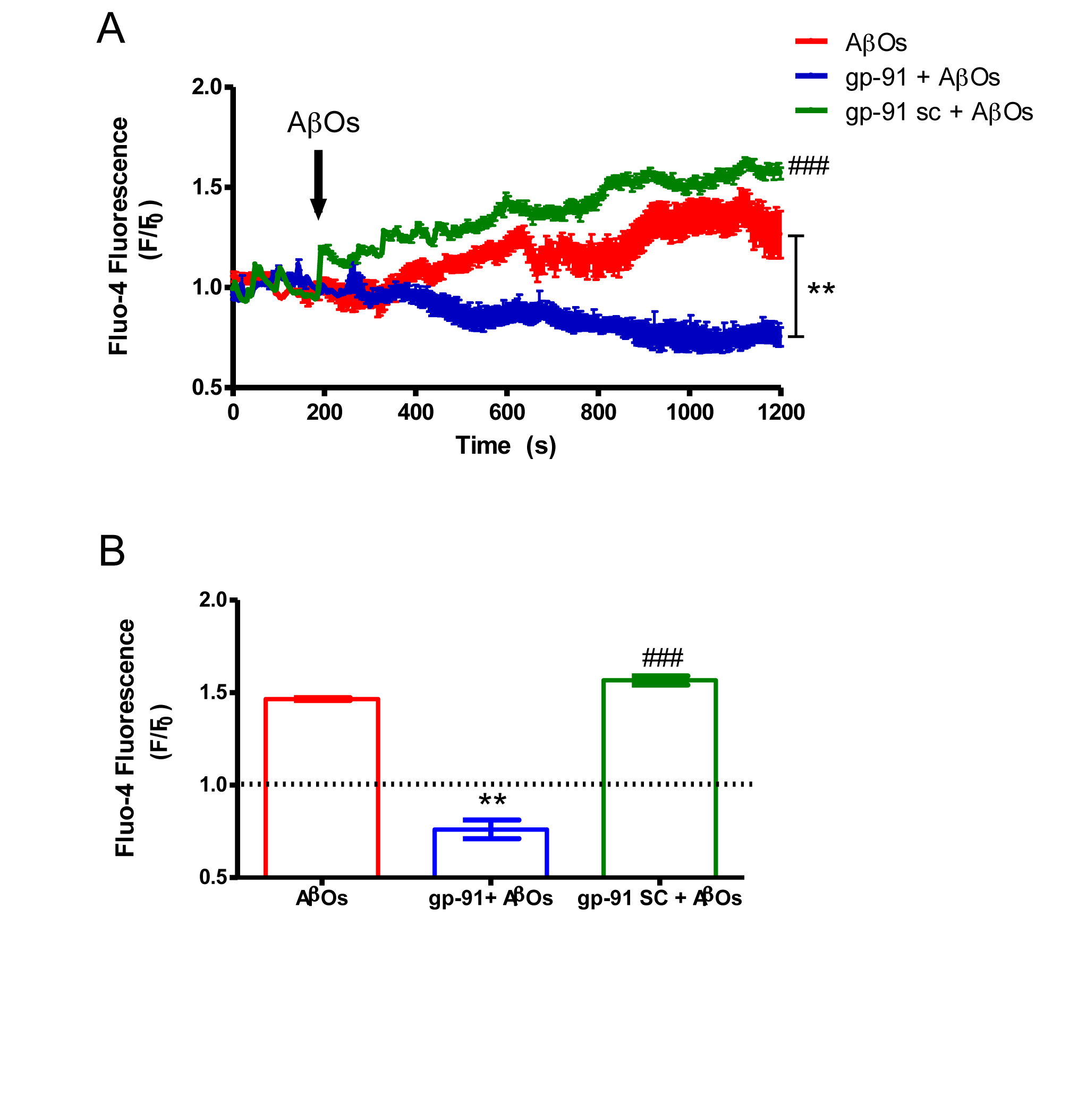

Supplement: Supplementary file 1 [file Image_1.TIF]

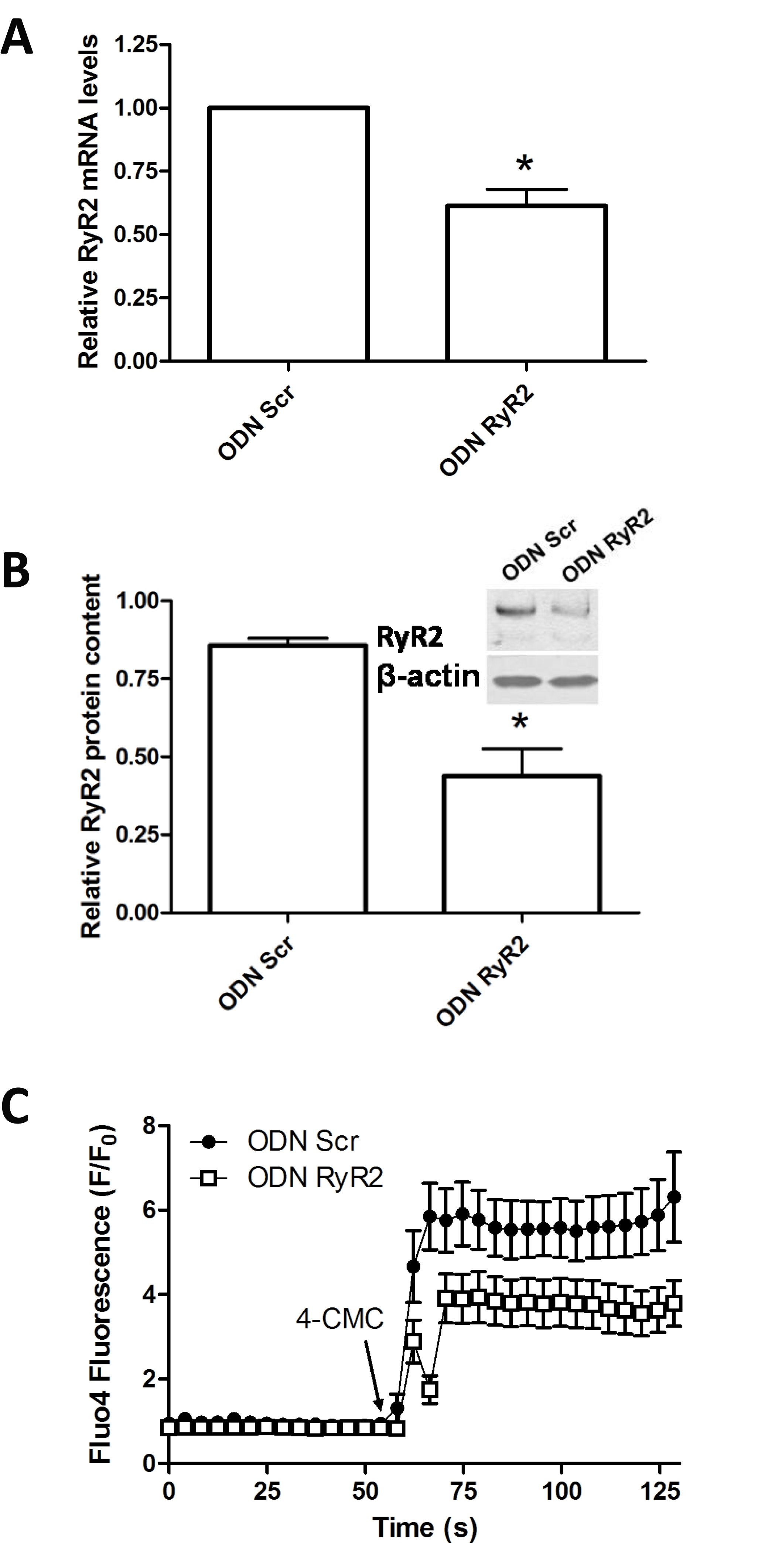

Supplement: Supplementary file 2 [file Image_2.TIF]

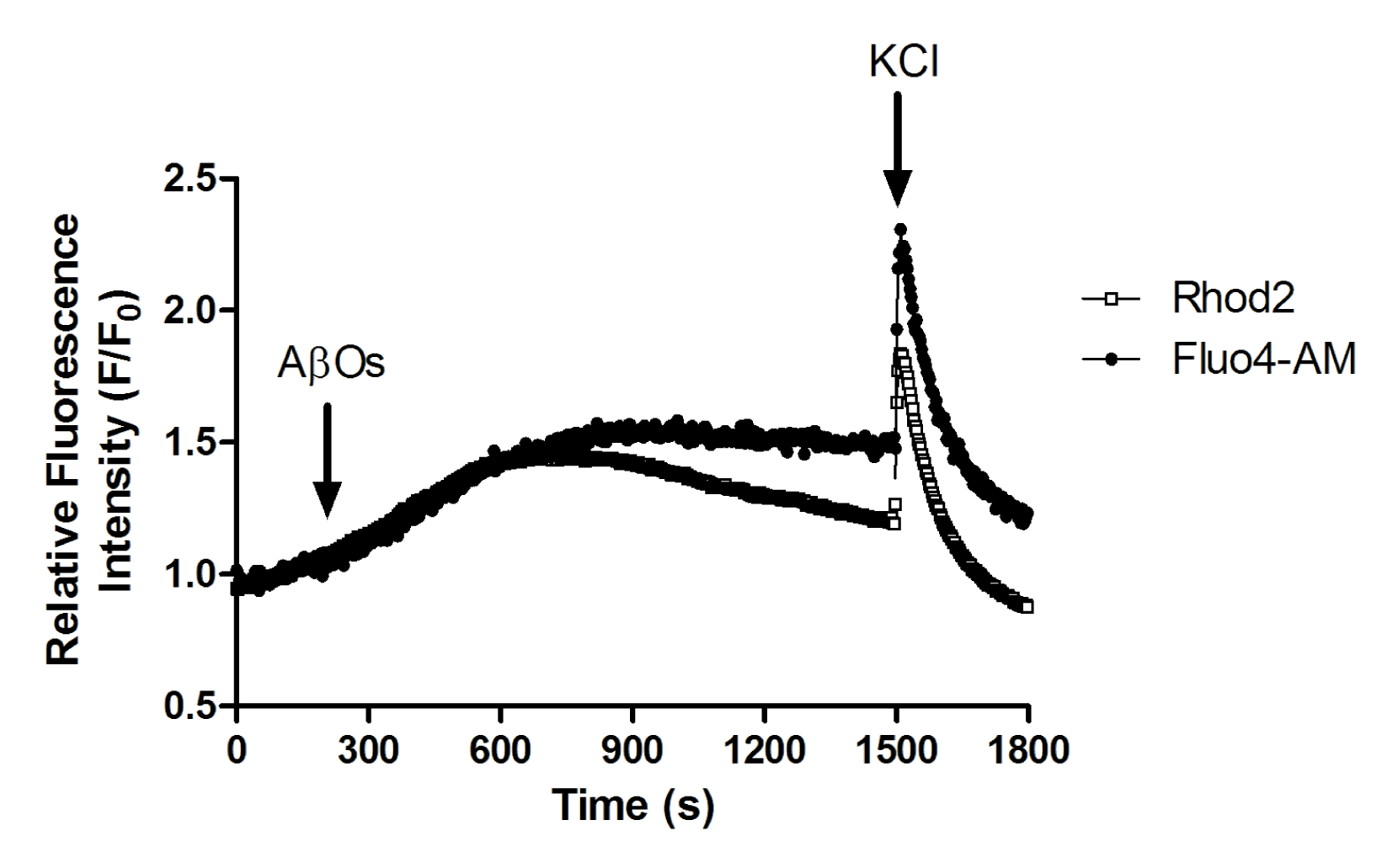

Supplement: Supplementary file 3 [file Image_3.TIF]
